# Efficacy of lung surfactant combined with budesonide in improving bronchopulmonary dysplasia and growth of very low birth weight infants

**DOI:** 10.3389/fped.2025.1686811

**Published:** 2025-11-26

**Authors:** Yu Zhang, Ming Liu, Yue Li, Di Chi, Jinpu Zhang, Yunfeng Zhang

**Affiliations:** Department of Neonatology, The Second Hospital of Jilin University, Changchun, Jilin, China

**Keywords:** budesonide suspension, pulmonary surfactant, clinical trial, local application, bronchopulmonary dysplasia

## Abstract

**Background:**

There is few treatments for bronchopulmonary dysplasia (BPD), and systemic glucocorticoid therapy has serious side effects.

**Methods:**

Low birth weight infants were classified randomly into control group administered with pulmonary surfactant (200 mg/kg) and intervention group administered with pulmonary surfactant (200 mg/kg) and budesonide suspension (0.25 mg/kg) to explore the efficacy of combination of pulmonary surfactant and budesonide suspension is better than that of pulmonary surfactant alone.

**Results:**

The incidence of bronchopulmonary dysplasia was significantly lower in the intervention group (45%) compared to the control group (64%). Additionally, the duration of invasive ventilator use was significantly shorter in the intervention group (66.39 ± 37.09 h) than in the control group (82.05 ± 54.55 h); and the infants from the intervention group had a significantly shorter supplemental oxygen time, with the intervention group at 775.32 ± 396.06 h and the control group at 844.01 ± 414.18 h. Comparison of the basic conditions of the two groups of children showed no statistically significant differences in maternal medical history, gestational age, birth weight, sex, whether hormones were used prenatally, and delivery method (*P* > 0.05). There was no difference in the incidence of complications, such as neonatal infection, intracranial hemorrhage, necrotizing enterocolitis, and retinopathy between the two groups.

**Conclusion:**

Combination of pulmonary surfactant with budesonide suspension can significantly decrease the incidence of bronchopulmonary dysplasia in very low birth weight infants, reduce the duration of invasive mechanical ventilation, and promote earlier weaning from oxygen supplement.

**Clinical Trial Registration:**

Effect of budesonide combined with pulmonary surfactant on lung development in very low birth weight infants, ChiCTR2400086677, https://www.chictr.org.cn/showproj.html?proj=233373.

## 1 Introduction

With the rapid advancement of perinatal medicine, the birth and survival rates of very low birth weight infants (VLBW, <1,500 g) and extremely low birth weight infants (ELBW, <1,000 g) have significantly increased. Although neonatal care has continuously improved, the incidence of bronchopulmonary dysplasia (BPD) continues to rise annually (1). BPD refers to preterm infants born before 32 weeks of gestation. Assessment is conducted at 36 weeks of corrected gestational age or at discharge. According to the 2019 Neonatal Research Network criteria, the classification is as follows: no BPD if no respiratory support is required; Grade I BPD if nasal cannula oxygen therapy is used at a flow rate ≤2 L/min; Grade II BPD if nasal cannula oxygen therapy is used at a flow rate >2 L/min or non-invasive mechanical ventilation is required; and Grade III BPD if invasive mechanical ventilation is needed (2). In China, BPD occurs in 74.2% of preterm infants born at ≤25 weeks' gestation, 51.9% at 26–27 weeks, 33.4% at 28–29 weeks, and 19.3% at 30–31 weeks (3).

Bronchopulmonary dysplasia (BPD) is a multifactorial disease primarily stemming from prematurity and low birth weight. Infants born preterm have immature lungs characterized by underdeveloped alveoli, thickened alveolar walls, and an insufficient pulmonary vasculature, which predisposes them to respiratory dysfunction and initial lung injury. Further contributing to the development of BPD are oxygen therapy and mechanical ventilation. Prolonged exposure to high-concentration oxygen can induce alveolar oxygen toxicity, while ventilator-induced barotrauma damages delicate lung tissue and disrupts alveolar architecture. Finally, prenatal or postnatal infections and associated inflammatory responses can trigger abnormal tissue proliferation and injury, thereby playing a significant role in the pathogenesis of BPD.

Neonatal respiratory distress syndrome (NRDS), caused by pulmonary surfactant (PS) deficiency, is effectively treated with exogenous PS. Large-scale clinical studies demonstrate that endotracheal PS administration improves lung compliance, reduces pulmonary vascular resistance, and enhances pulmonary blood flow (4).

Budesonide, a potent topical glucocorticoid, undergoes extensive hepatic metabolism into low-activity metabolites. It enhances lung compliance, reduces transcutaneous CO₂, promotes ciliary function, decreases microvascular permeability, and mitigates inflammation, thereby improving pulmonary ventilation (5).

Recent studies highlight the combined use of budesonide and porcine-derived PS for NRDS and BPD management. Intratracheal co-administration improves oxygenation, alleviating respiratory distress; reduces ventilator/oxygen dependence, enabling earlier weaning and lowering complication risks; lowers BPD incidence by attenuating lung inflammation and promoting normal development. A meta-analysis by Yu Jialin et al. confirmed that PS-budesonide combination therapy reduces BPD incidence and mortality without increasing short-term complications (6).

Thus, this study aims to evaluate the efficacy of budesonide combined with porcine PS in promoting lung development and preventing BPD in VLBW infants.

## 2 Materials and methods

### 2.1 Participants

200 preterm newborns with a gestational age of less than 32 weeks, birth weight less than 1,500 g, and diagnosed with NRDS admitted to the Neonatal Intensive Care Unit (NICU) of the Second Hospital of Jilin University from January 2022 to December 2023 were selected as the study participants. The study was approved by the Institutional Review Board of the Second Hospital of Jilin University and was conducted in accordance with the tenets of the Declaration of Helsinki. All parents of patients who participated in the trial provided informed consent before inclusion.

### 2.2 Inclusion and exclusion criteria

The diagnostic criteria for NRDS meet the standards of the 4th edition of Practical Neonatology (7). Inclusion criteria: (1) infants requiring mechanical ventilation within 4 h after birth due to severe NRDS; (2) initial need for inhaled oxygen concentration ≥40%. Exclusion criteria: (1) asphyxia after birth, with or without organ damage; (2) comorbid surgical diseases or congenital heart disease; (3) congenital metabolic disorders.

### 2.3 Group composition

This study was designed as a double-blind, randomized controlled trial. The randomization sequence was generated by an independent statistician using SPSS. Participants were enrolled and assigned to either the control or intervention group by the staff from the Department of Nursing Management, who were not involved in the subsequent clinical assessment or data analysis. The interventions (porcine pulmonary surfactant and budesonide suspension, provided by Chiesi Farmaceutici S.p.A, Italy) were prepared and administered by this same department to ensure allocation concealment. All other investigators, clinicians, and outcome assessors were blinded to the group assignments throughout the study.

The sample size was determined based on previous studies, in which the rate of bronchopulmonary dysplasia in preterm infants was approximately 80% (8). We considered a reduction to 60% following the intervention to be clinically meaningful. With *α* = 0.05 and *β* = 0.2 (power = 80%), the calculated sample size was 79, using the formula below. Accounting for a dropout rate of 25%, a total of 100 participants were enrolled in the present trial.

$$N\;=\;\frac{\left[Z_{1\text{-}\alpha /2}\sqrt{2\bar{P}(1-\bar{P})}\;+\;Z_{\beta }\sqrt{P_{1}(1-P_{1})\;+\;P_{2}(1-P_{2})}\right]}{P_{1}-P_{2}}$$

Where, P_1_: Incidence rate in the control group; P_2_: Incidence rate in the intervention group; P: Average incidence rate, calculated as (P_1_ + P_2_)/2; Z₁-α/₂: The standard normal deviate corresponding to a two-sided significance level of α; Zβ: The standard normal deviate corresponding to a power of 1-β. N: The calculated sample size required for each group.

### 2.4 Instrument

Upon admission, all the participants were given invasive mechanical ventilation (tracheal intubation). Control group: Only pulmonary surfactant (Curosurf, poractant alfa, Chiesi Farmaceutici S.p.A, Italy) was administered to the participants at the dosage of 200 mg/kg. PS was instilled into the trachea of the participants within 1 h of life (the PS was preheated and maintained at body temperature level). Intervention group: Budesonide suspension 0.25 mg/kg and PS 200 mg/kg were instilled together into the trachea of the patients within 1 h of birth. The drug was instilled in three positions, supine position, left lateral decubitus position, right lateral decubitus position. Each instillation was performed with a 5-min interval between position changes (9).

The data on the following outcomes were collected and compared between control and intervention groups, the occurrence of bronchopulmonary dysplasia, the total duration of invasive mechanical ventilation and supplemental oxygen therapy, weight percentiles at corrected gestational age of 40 weeks, birth weight percentiles, complications, such as severe intracranial hemorrhage, neonatal infections, retinopathy of prematurity, and necrotizing enterocolitis during hospitalization.

### 2.5 Statistical analysis

SPSS 21.0 software was used to conduct statistical analysis. For continuous variables, the results were presented as mean ± standard deviation (SD), and ANOVA was employed to perform the comparisons between two groups, followed by a Bonferroni correction to adjust for multiple testing. For enumerated variables, χ^2^ test was used to conduct the comparisons. A *p-*value of <0.05 was considered statistically significant.

## 3 Results

### 3.1 General profile of the participants

**Figure 1:**
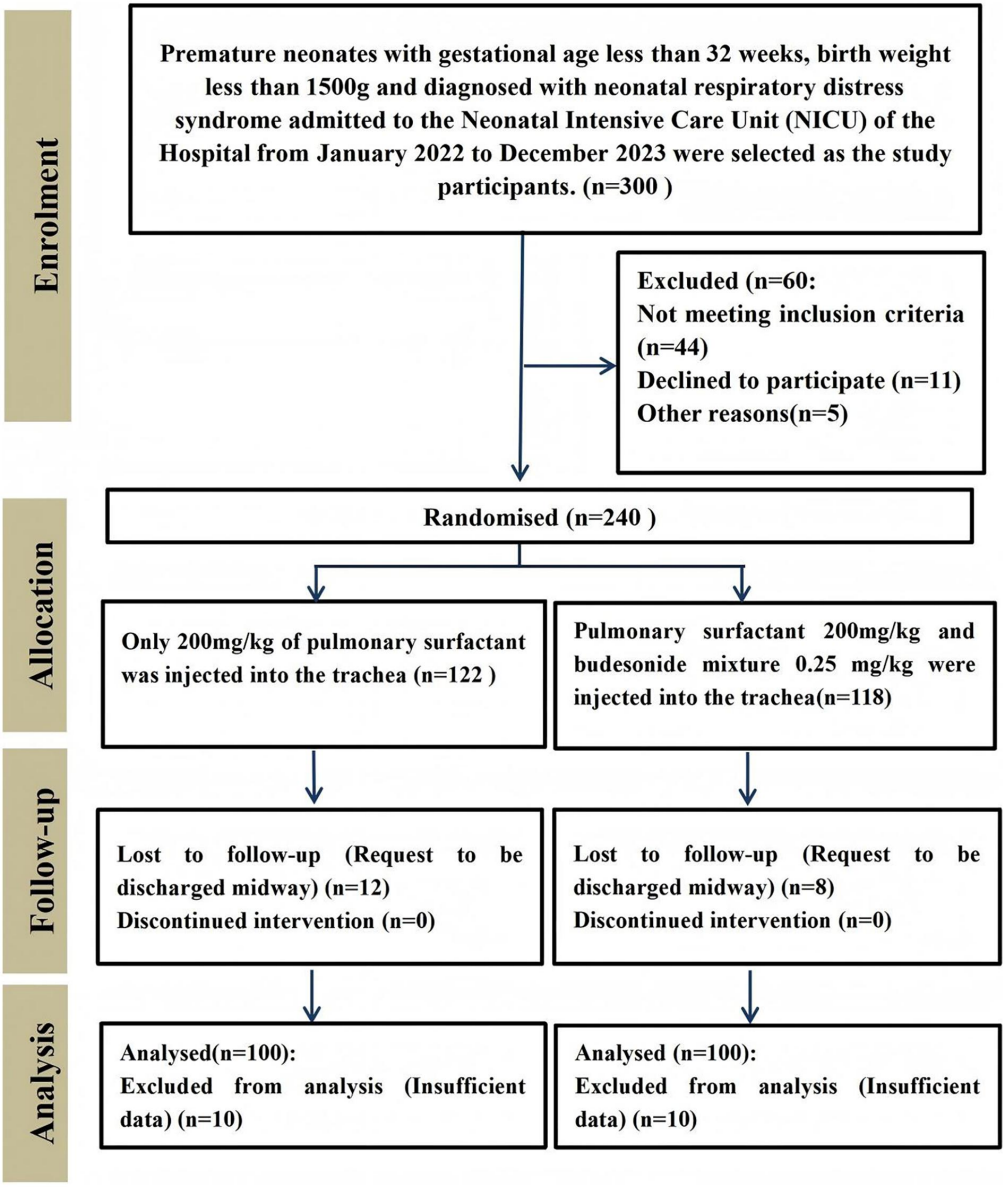
Participant enrollment process.

The general characteristics of the participants in each group were shown in Table 1, and there were no statistically significant differences in birth weight, gestational age and number of female and male patients between the two groups (*p* > 0.05, Table 1). The enrollment and assignment process were shown in Figure 1.

**Table 1:** Baseline characteristics of the participants (X ± s, 100).

Variables	Control group (100)	Intervention group (100)	*P*
Birth weight, kg	1.093 ± 0.24	1.095 ± 0.23	0.958
Gestational age, w	28.4 ± 1.88	28.7 ± 1.80	0.275
Number of male participants	54	53	0.887
Cesarean Section	35	27	0.221
Maternal GBS infection status	14	9	0.268
Antenatal steroid administration	79	84	0.363

Maternal GBS infection: Pregnant women diagnosed with Group B Streptococcus (GBS) infection either before or after delivery at our hospital's obstetrics department.

Antenatal corticosteroids: Administration of 2–4 doses of dexamethasone (or equivalent) to the mother prior to delivery to enhance fetal lung maturation.

### 3.2 Duration of invasive ventilator and supplemental oxygen use

The participants from the intervention group had significantly shorter duration of invasive ventilator and supplemental oxygen use compared with those from the control group (*P* < 0.05). The comparison of duration of invasive ventilator and supplemental oxygen use, and length of total hospitalization was shown in Table 2.

**Table 2:** Comparison of ventilator and oxygen use and length of total hospitalization in the participants between two groups (*N* = 100).

Variables	Control group (100)	Intervention group (100)	*P*
Ventilator use, hour	82.05 ± 54.55	66.39 ± 37.09	0.019
Oxygen use, hour	844.01 ± 414.18	775.32 ± 396.06	0.030
Length of total hospitalization, day	45.84 ± 16.75	42.36 ± 16.09	0.143

### 3.3 Bronchopulmonary dysplasia and complications

The incidence of bronchopulmonary dysplasia (BPD) was significantly lower in the intervention group than that in the control group (*P* < 0.05). There was no significant difference in the incidence of intracranial hemorrhage (IH), neonatal infection (NF), necrotizing enterocolitis (NEC), and retinopathy of prematurity (ROP) between the two groups of patients (*P* > 0.05), as shown in Table 3.

**Table 3:** Comparison of incidence of bronchopulmonary dysplasia and complication.

Variables	Control group (%)	Intervention group (%)	*P*
Bronchopulmonary dysplasia	64	45	0.007
Intracranial hemorrhage	54	56	0.776
Neonatal infection	44	53	0.275
Necrotizing enterocolitis	8	6	0.579
Retinopathy of prematurity	34	36	0.767
Requirement for rescue surfactant	24	16	0.157

Neonatal Infection: An infectious disease occurring within 28 days after birth, caused by invasion of various pathogens (e.g., bacteria, viruses), manifested by clinical symptoms and abnormal laboratory findings.

Intracranial Hemorrhage: Hemorrhagic events occurring at different intracranial locations in neonates, diagnosed by cranial ultrasound findings.

Necrotizing Enterocolitis (NEC): A life-threatening gastrointestinal emergency characterized by intestinal necrosis secondary to infection or other causes, confirmed by clinical manifestations and diagnostic tests.

Retinopathy of Prematurity (ROP): A retinal vascular developmental disorder in preterm infants caused by multiple factors, diagnosed through fundus examination.

Criteria for Rescue Pulmonary Surfactant Administration, decision is based on: persistent respiratory distress unrelieved by initial treatment, inability to maintain normal transcutaneous oxygen saturation despite high ventilator settings, reduced lung radiolucency on chest DR imaging, exclusion of other causes of respiratory distress.

### 3.4 Comparison of *z* scores

There was no difference in *z* scores at birth and corrected gestational age of 40 weeks between the two groups (Table 4).

**Table 4:** Comparison of *z* scores at birth and corrected gestational age at of weeks.

Variables	Control group (100)	Intervention group (100)	*P*
*Z* scores at birth	−0.11 ± 1.15	0.18 ± 1.26	0.085
*Z* scores at corrected gestational age of 40 weeks	0.92 ± 0.96	1.05 ± 1.19	0.401

*Z*-scores at birth and at a corrected gestational age of 40 weeks: (Actual body weight - Median weight for the same sex and gestational age)/Standard deviation of weight for the same sex and gestational age.

## 4 Discussion

In the treatment process of preterm infants, those who suffer from severe respiratory distress face a crucial issue after long-term mechanical ventilation therapy. Namely, the lungs of preterm infants are not fully developed. After being injured by mechanical ventilation and high concentrations of oxygen, the lung tissue structure is restructured, a large amount of fibrous tissue shows hyperplasia, the basal membrane of the alveolar and capillary septum thickens, the number of alveoli decreases, the structure simplifies, and the function further decreases (10). This leads to an increase in the resistance of pulmonary ventilation, a decrease in compliance, and gradually evolves into BPD, causing difficulties in weaning from ventilator and oxygen cessation. It also results in slow growth of preterm infants, or even growth stagnation, severely affecting the development of brain nerves (10). Therefore, shortening the duration of mechanical ventilation and supplemental oxygen in preterm infants is crucial in preventing the occurrence of BPD.

Currently, there is still no effective treatment for BPD. The primary causes of classic BPD are mechanical ventilation injury and oxygen toxicity. These infants are mostly preterm babies with a gestational age over 32 weeks who, due to severe respiratory distress syndrome (RDS), require high-concentration oxygen therapy and high airway pressure mechanical ventilation, leading to direct lung tissue damage and abnormal repair. The etiology of the new form of BPD is more complex, primarily involving pulmonary immaturity and inflammatory responses. These infants are mostly extremely premature with a gestational age under 26 weeks, and their pathogenesis involves interactions among multiple factors, including arrested lung development, intrauterine infection or inflammation, oxygen toxicity, and mechanical ventilation injury (11).

Glucocorticoids have once been used as conventional drugs for the prevention and treatment of BPD (12). Glucocorticoids can promote the synthesis of PS and the generation of lung antioxidant enzymes, reduce pulmonary inflammatory responses, inhibit inflammatory cell infiltration, producing a therapeutic effect on BPD (12). However, with the in-depth clinical research, many studies have successively reported adverse reactions to the systemic use of glucocorticoids (13). As reported by Doyle LW et al., early systemic corticosteroid use in preterm infants can overall reduce the incidence of BPD at 36 weeks of corrected gestational age. However, it is associated with an increased risk of gastrointestinal perforation and cerebral palsy, along with other significant adverse effects including infection, elevated blood pressure, hyperglycemia, impaired head growth, and a higher incidence of periventricular leukomalacia (14). Therefore, the current guidelines of the American and Canadian Pediatric Societies do not recommend routine systemic glucocorticoid use for preventing and treating BPD in very low birth weight infants (15). Similarly, the European Society of Perinatal Medicine guidelines recommend avoiding the use of glucocorticoids as much as possible (16). Current research indicates that the increasing number of adverse reactions caused by systemic glucocorticoid use has led researchers to pay more attention to the local application of glucocorticoids. The study by Pan et al. showed that compared with intratracheal administration of PS alone, the combination of PS and budesonide via intratracheal instillation significantly improved ventilation and gas exchange functions in very low birth weight preterm infants with severe respiratory distress syndrome complicated by infection and inflammation. It also markedly reduced the incidence of BPD, with no difference in short-term complications or mortality (17).

Ye et al. (18) demonstrated that early, targeted, low-dose pulmonary corticosteroid therapy is a highly promising strategy for preventing BPD, without increasing the risk of short-term complications such as sepsis, intraventricular hemorrhage, or retinopathy of prematurity. Their study confirmed the efficacy and safety of endotracheal administration of budesonide combined with pulmonary surfactant. Similarly, the study by Bassler et al. (19) further substantiated that early inhalation of budesonide in extremely preterm infants significantly reduces the incidence of BPD, though this benefit must be weighed against a potential increase in mortality risk.

Additionally, it is also needed to highlight that surfactant improves budesonide solubilization, facilitates absorption, and supports conjugation with fatty acids.

The results of the present study showed that after treatment with Budesonide and PS, the total duration of invasive mechanical ventilation and oxygen therapy during hospitalization in the intervention group was significantly shorter than that in the control group (*P* < 0.05). This indicates that the intervention had a more positive effect on respiratory recovery time and BPD incidence, without increasing the rates of complications such as intracranial hemorrhage, neonatal infection, retinopathy of prematurity, or neonatal necrotizing enterocolitis. Furthermore, there was no significant difference in the *Z*-scores at 40 weeks of corrected gestational age between the control group and the intervention group, suggesting no significant impact on the growth and development of preterm infants. Regarding the crucial concern of whether it increases the risk of neurological impairment, longer-term follow-up and further research are still required (20).

This study has limitations, including its single-center design, small sample size, no long-term neurodevelopmental follow-up and no proactive monitor for hypoglycaemia, gastric bleeding, or hypertension as predefined endpoints. Moreover, with advances in medical care, the incidence of BPD in preterm infants with a gestational age >28 weeks has gradually declined. Future research should focus on extremely preterm infants (<28 weeks) and involve larger, multicenter studies for further validation.

## 5 Conclusion

The combination of budesonide and pulmonary surfactant effectively reduces the duration of mechanical ventilation and oxygen dependency in very low birth weight infants, contributing to the prevention of BPD, improved respiratory function, and catch-up growth in weight. However, it showed no significant impact on total hospitalization length, the need for additional PS supplementation, or the incidence of complications such as neonatal infections, intracranial hemorrhage, necrotizing enterocolitis, or retinopathy of prematurity.

## Data Availability

The original contributions presented in the study are included in the article/Supplementary Material, further inquiries can be directed to the corresponding author.

## References

[1] Nakashima T Inoue H Sakemi Y Ochiai M Yamashita H Ohga S Trends in bronchopulmonary dysplasia among extremely preterm infants in Japan, 2003–2016. J Pediatr. (2021) 230:119–25.e7. 10.1016/j.jpeds.2020.11.041 33246013

[2] Jensen EA Dysart K Gantz MG McDonald S Bamat NA Keszler M The diagnosis of bronchopulmonary dysplasia in very preterm infants. An evidence-based approach. Am J Respir Crit Care Med. (2019) 200(6):751–9. 10.1164/rccm.201812-2348OC 30995069 PMC6775872

[3] Li R Huang D. Research progress on single-cell sequencing technology in bronchopulmonary dysplasia. Chin J Neonatol (Chin Eng). (2023) 38(11):701–4.

[4] Neonatology Group of Pediatric Society, Chinese Medical Association; Editorial Board of Chinese Journal of Pediatrics. Expert consensus on the clinical application of pulmonary surfactant in neonates in China (2021 edition). Chin J Pediatr. (2021) 59(8):627–32.

[5] Liu MM Ji L Dong MY Zhu XF Wang HJ. A prospective randomized controlled trial of endotracheal administration of budesonide combined with pulmonary surfactant for the prevention of bronchopulmonary dysplasia. Chin J Contemp Pediatr. (2022) 24(1):78–84. 10.7499/j.issn.1008-8830.2109106 PMC8802381 35177180

[6] Guo YY Chen H Yu JL. Efficacy and safety of intratracheal administration of pulmonary surfactant combined with budesonide for preventing bronchopulmonary dysplasia: a meta-analysis. J Pediatr Pharm. (2019) 25(5):1–5.

[7] Shao XM Ye HM Qiu XS. Practical Neonatology. 4th ed. Beijing: People’s Medical Publishing House (2013). p. 395–8.

[8] Zhu Z Yuan L Wang J Li Q Yang C Gao X Mortality and morbidity of infants born extremely preterm at tertiary medical centers in China from 2010 to 2019. JAMA Netw Open. (2021) 4(5):e219382. 10.1001/jamanetworkopen.2021.9382 33974055 PMC8114138

[9] Ke H Li ZK Yu XP Guo JZ. Comparison of the efficacy of different budesonide formulations combined with pulmonary surfactant in the treatment of neonatal respiratory distress syndrome. Chin J Contemp Pediatr. (2016) 18(5):400–4. 10.7499/j.issn.1008-8830.2016.05.005 PMC7390364 27165587

[10] Carvalho CG Silveira RC Procianoy RS. Ventilator-induced lung injury in preterm infants. Rev Bras Ter Intensiva. (2013) 25(4):319–26. 10.5935/0103-507X.20130054 24553514 PMC4031878

[11] Kalikkot Thekkeveedu R Guaman MC Shivanna B. Bronchopulmonary dysplasia: a review of pathogenesis and pathophysiology. Respir Med. (2017) 132:170–7. 10.1016/j.rmed.2017.10.014 29229093 PMC5729938

[12] Doyle LW Cheong JL Hay S Manley BJ Halliday HL. Early (<7 days) systemic postnatal corticosteroids for prevention of bronchopulmonary dysplasia in preterm infants. Cochrane Database Syst Rev. (2021) 10(10):CD001146. 10.1002/14651858.CD001146.pub6 34674229 PMC8530019

[13] Committee on Fetus and Newborn. Postnatal corticosteroids to treat or prevent chronic lung disease in preterm infants. Pediatrics. (2002) 109(2):330–8. 10.1542/peds.109.2.330 11826218

[14] Szabó H Baraldi E Colin AA. Corticosteroids in the prevention and treatment of infants with bronchopulmonary dysplasia: part I. Systemic corticosteroids. Pediatr Pulmonol. (2022) 57(3):600–8. 10.1002/ppul.25805 34964559

[15] Onland W Offringa M van Kaam A. Late (≥7 days) inhalation corticosteroids to reduce bronchopulmonary dysplasia in preterm infants. Cochrane Database Syst Rev. (2017) 8(8):CD002311. 10.1002/14651858.CD002311.pub4. Update in: Cochrane Database Syst Rev. (2022) 12:CD002311. doi: 10.1002/14651858.CD002311.pub5. 28836266 PMC6483527

[16] Sweet DG Carnielli VP Greisen G Hallman M Klebermass-Schrehof K Ozek E European Consensus guidelines on the management of respiratory distress syndrome: 2022 update. Neonatology. (2023) 120(1):3–23. 10.1159/000528914 36863329 PMC10064400

[17] Pan J Chen MW Ni WQ Fang T Zhang H Chen Y Therapeutic effect of pulmonary surfactant combined with budesonide in preventing bronchopulmonary dysplasia in very low birth weight infants. Chin J Contemp Pediatr. (2017) 19(2):137–41. 10.7499/j.issn.1008-8830.2017.02.002 PMC7389465 28202108

[18] Yeh TF Chen CM Wu SY Husan Z Li TC Hsieh WS Intratracheal administration of budesonide/surfactant to prevent bronchopulmonary dysplasia. Am J Respir Crit Care Med. (2016) 193(1):86–95. 10.1164/rccm.201505-0861OC 26351971

[19] Bassler D Shinwell ES Hallman M Jarreau PH Plavka R Carnielli V Neonatal European study of inhaled steroids trial group. Long-term effects of inhaled budesonide for bronchopulmonary dysplasia. N Engl J Med. (2018) 378(2):148–57. 10.1056/NEJMoa1708831 29320647

[20] Perrone S Orlando S Petrolini C Marinelli F Moretti S Corradi M Current concepts of corticosteroids use for the prevention of bronchopulmonary dysplasia. Curr Pediatr Rev. (2023) 19(3):276–84. 10.2174/1573396318666220804100251 36043724

